# Patients’ perspectives and preferences toward telemedicine versus in-person visits: a mixed-methods study on 1226 patients

**DOI:** 10.1186/s12911-023-02348-4

**Published:** 2023-11-15

**Authors:** Khadijeh Moulaei, Abbas Sheikhtaheri, Farhad Fatehi, Mostafa Shanbehzadeh, Kambiz Bahaadinbeigy

**Affiliations:** 1 Department of Health Information Technology, School of Paramedical, Ilam University of Medical Sciences, Ilam, Iran; 2 Department of Health Information Management, School of Health Management and Information Sciences, Iran University of Medical Sciences, Tehran, Iran; 3 School of Business, The University of Queensland, Brisbane, Australia; 4 Department of Health Information Management and Technology, Medical Informatics Research Center, Institute for Futures Studies in Health, Kerman University of Medical Sciences, Kerman, Iran

**Keywords:** Telemedicine, Telehealth, Patient preference

## Abstract

**Introduction:**

Despite the fact that telemedicine can eliminate geographical and time limitations and offer the possibility of diagnosing, treating, and preventing diseases by sharing reliable information, many individuals still prefer to visit medical centers for in-person consultations. The aim of this study was to determine the level of acceptance of telemedicine compared to in-person visits, identify the perceived advantages of telemedicine over in-person visits, and to explore the reasons why patients choose either of these two types of visits.

**Methods:**

We developed a questionnaire using the rational method. The questionnaire consisted of multiple-choice questions and one open-ended question. A total of 2059 patients were invited to participate in the study. Chi-square tests and descriptive statistics were employed for data analysis. To analyze the data from the open-ended question, we conducted qualitative content analysis using MAXQDA 18.

**Results:**

Out of the 1226 participants who completed the questionnaire, 865 (71%) preferred in-person visits, while 361 (29%) preferred telemedicine. Factors such as education level, specific health conditions, and prior experience with telemedicine influenced the preference for telemedicine. The participants provided a total of 183 different reasons for choosing either telemedicine (108 reasons) or in-person visits (75 reasons). Avoiding infectious diseases, saving cost, and eliminating and overcoming geographical distance barriers were three primary telemedicine benefits. The primary reasons for selecting an in-person visit were: more accurate diagnosis of the disease, more accurate and better examination of the patient by the physician, and more accurate and better treatment of the disease.

**Conclusion:**

The results demonstrate that despite the numerous benefits offered by telemedicine, the majority of patients still exhibit a preference for in-person visits. In order to promote broader acceptance of telemedicine, it becomes crucial for telemedicine services to address patient preferences and concerns effectively. Employing effective change management strategies can aid in overcoming resistance and facilitating the widespread adoption of telemedicine within the population.

**Supplementary Information:**

The online version contains supplementary material available at 10.1186/s12911-023-02348-4.

## Introduction

Geographical and time limitations prevent access to healthcare in cities, suburbs, and villages. Telemedicine can overcome many healthcare access problems [1]. Telemedicine enables the diagnosis, treatment, and prevention of diseases by facilitating the sharing of reliable information through information and communication technologies [2]. Telemedicine refers to the provision of remote clinical services via synchronous or asynchronous two-way communication between the patient and the healthcare provider, using information and communication technologies [3]. In the synchronous mode (such as video conferences or telephone), patients can engage in real-time communication with their doctors, enabling immediate diagnosis and treatment recommendations. In the asynchronous or store-and-forward mode, patients can share medical images, test results, or descriptions of symptoms, allowing healthcare providers to review the information at a later time and respond with appropriate guidance or prescriptions [4, 5]. Telemedicine, through these two modes, has the potential to offer patients convenient access to healthcare [6]. Moreover, telemedicine offers tremendous potential to lower healthcare costs and enhance access to various care services for underserved populations [7]. Despite the many benefits of telemedicine, many people seek medical services through in-person visits for various reasons [7–11].

The participants in the Jansen-Kosterink study [12] stated that a lack of intrinsic motivation in patients, impersonal communication, technical issues, digital literacy concerns, privacy infringements, potential for additional complaints, perceived lower treatment intensity, and difficulty ensuring up-to-date content can cause patients to reject or be less accepting of telemedicine. Moreover, the demand for in-person visits may vary based on the patient’s demographics and co-morbidities. Jabbarpour et al. [13] indicated that patients with chronic diseases such as hypertension and coronary artery disease, elderly patients, and non-Hispanic Black patients are less likely to use telemedicine due to factors such as distrust in the use of technology for healthcare, poor health literacy, and low technology literacy [14, 15] and concerns related to poor clinical outcome [7], perceived misdiagnosis of diseases, ethical and legal issues, patient privacy, information confidentiality, liability, and potential problems like negligence and malpractice in the use of telemedicine contribute to the preference for in-person visits [16]. However, the difference in access to technology also plays an important role in choosing telemedicine or in-person visits [13]. For example, some studies have indicated that Black and Hispanic patients are less likely than non-Hispanic White patients to own a smartphone or have access to broadband Internet [17]. Furthermore, a survey conducted within the Kaiser Permanente system revealed that older patients (aged over 75) as well as Black, Latino, and Filipino patients were less likely to possess digital devices and also less inclined to use the Internet and electronic devices [10]. The Kaiser Permanente system is a large integrated healthcare organization in the United States that provides health insurance coverage along with healthcare services through its network of hospitals, clinics, and medical facilities. It emphasizes preventive care, patient-centered approaches, and electronic health records for comprehensive healthcare delivery [18].

To our knowledge, many studies are available to examine the perspectives of patients regarding telemedicine versus in-person visits. Most of the studies conducted so far have focused solely on patients with specific diseases (such as COVID-19, Parkinson’s disease, heart diseases, multiple sclerosis) [17–20] or disabilities (like knee arthroplasty) [19–22] or disabilities (like knee arthroplasty) [23] in relation to their preference for telemedicine or in-person visits. Moreover, in many of these studies, the factors influencing the choice between telemedicine and in-person visits have not been thoroughly identified. Therefore, this study was designed to achieve three key objectives. Firstly, it seeks to gauge the extent of patient acceptance regarding telemedicine in contrast to conventional in-person visits. Secondly, it aims to pinpoint the perceived benefits associated with telemedicine over in-person visits. Lastly, the study endeavors to delve into the underlying factors that drive patients to opt for either telemedicine or in-person visits.

## Methods

### Study population

**Fig. 1:**
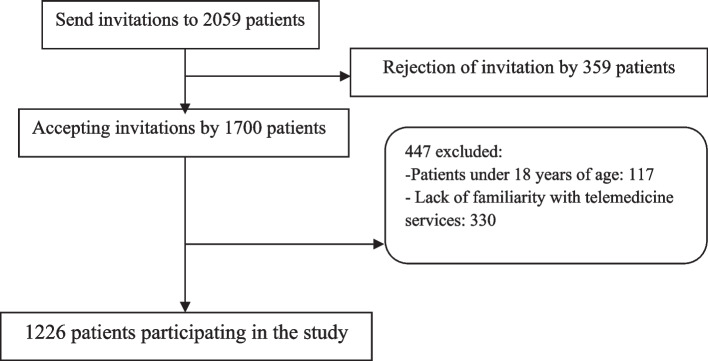
Study recruitment flow diagram

The study population comprised patients referred to medical centers and hospitals in four western provinces of Iran: Khuzestan, Lorestan, Kermanshah, and Ilam. Upon presenting letters of introduction to the medical centers and hospitals within these provinces, the researchers were provided with contact information (phone numbers) for 2059 patients. Invitation letters were sent to these patients through social networks using platforms such as WhatsApp and Telegram, inviting them to participate in the study. Among the invitations, one thousand seven hundred patients accepted, and ultimately, based on the inclusion criteria outlined below, 1226 patients were selected for the study (refer to Fig. 1).

- Familiarity with telemedicine services
- Age of at least 18 years
- Previous admission to a health center or hospital
- Provision of informed consent

### Questionnaire development

In this study, we designed a questionnaire using a hybrid approach that incorporates insights from literature reviews, expert consultations, and collaborative team discussions [24]. The questionnaire was designed by reviewing relevant studies [2, 13, 25–31] and consulting with three experts in medical informatics and health information management, each possessing at least five years of scientific and research experience in the field of telemedicine. To create this questionnaire, the research team held three four-hour meetings. As a result, the questionnaire was divided into four sections. The first part included demographic information (consisting of 4 demographic questions), and the second part comprised four questions related to the type of disease, its duration, and the history of using telemedicine. The third part encompassed questions about the benefits of using telemedicine compared to in-person visits (a total of 36 questions). In this study, the benefits of telemedicine encompass a set of advantageous factors that streamline the provision of essential healthcare services. It facilitates not only patients’ access to care at anytime and anywhere but also healthcare providers’ delivery of necessary treatments, ultimately enhancing the overall efficiency and effectiveness of the healthcare system. The fourth section posed the question to participants: ‘What is your preference – telemedicine or an in-person visit?’ Following that, an open-ended question prompted them to explain the reasons for their selection. The questions pertaining to the demographic and clinical information of the participants were presented in a multiple-choice format, while the questions regarding the ‘advantages of using telemedicine versus in-person visits’ were structured as a 5-point Likert scale (see Additional file 1).

The face and content validity of the questionnaire were verified by four medical informatics experts (who have a history of engaging in scientific research activities related to telemedicine). The questionnaire was then converted into an electronic format using Google Forms. To mitigate the occurrence of missing responses, all questions in the questionnaire were marked as mandatory. After conducting a pilot test involving 100 patients, the reliability of the questionnaire was assessed. The values obtained were 0.991 and 0.81 for four-option and two-option questions, respectively, calculated through Cronbach’s alpha and Kuder–Richardson tests (see Additional file 1).

Demographic information (section A) and medical conditions (section B) are typically not included in the calculation of Cronbach’s alpha because they are not part of the scale meant to measure a specific construct. This information is collected to describe the characteristics of your study participants, while Cronbach’s alpha is used to assess the reliability of the scale itself [32]. Therefore, Cronbach’s alpha was computed for all 36 questions in section C and the first question of section D.

### Data collection

Prior to distributing the questionnaire to the participants, 30 test users completed the questionnaire online to accurately gauge the time required for its completion. These users recorded both the start and finish times. The time needed to complete the questionnaire ranged from 10 to 15 min. The questionnaire link was distributed to the patients through social networks, specifically WhatsApp and Telegram, from October 3 to October 30, 2021. By November 30, 2021, the participants had responded to the questionnaires. In addition to the link, participants received a guide on how to complete the questionnaire. This guide included contact information, such as the phone number of one of the researchers, so that participants could reach out for assistance if necessary.

It should be noted that to assess participants’ familiarity with telemedicine services, we included a screening question before participants entered the questionnaire. Participants were asked if they were familiar with telemedicine interventions and its concepts. Those who responded affirmatively were considered knowledgeable and eligible to proceed with the questionnaire, after which they were seamlessly directed to the online survey. In contrast, participants who indicated that they were unfamiliar with telemedicine received a message titled ‘You are not authorized to complete this questionnaire’ and were subsequently excluded from further participation. It’s important to note, however, that some participants who claimed familiarity with telemedicine may not have had prior experience with it, as indicated in the results section (Table 1).

**Table 1:** Demographic and clinical characteristics of the participants

**Variables**	**Variable type**	**Frequency**	**Percentage**
**Sex**	Male	304	24.8
	Female	922	75.2
**Age**	18–27	661	53.9
	28–37	370	30.2
	38–47	159	13.0
	48–57	29	2.4
	> 57	7	0.6
**Education level**	Diploma	441	36
	Associate degree	103	8.4
	Bachelor	480	39.2
	Master	156	12.6
	PhD	46	3.8
**Residence**	Metropolitan areas	1105	90.1
	Remote and rural areas	121	9.9
**Disease type**	Oral and dental diseases	359	29.3
	Eye diseases	291	23.8
	Skin and hair diseases	281	22.9
	Digestive diseases	254	20.7
	Otorhinolaryngology diseases	190	10.5
	Gynecological diseases	140	11.4
	Endocrine diseases	121	9.9
	Psychological disorders	101	8.2
	Musculoskeletal diseases	95	7.7
	Cardiovascular diseases	85	6.9
	Respiratory diseases	61	5
	Cancers	10	0.8
	Rheumatology diseases	28	2.3
	Kidneys and urinary diseases	73	6
	Genetic disorders	18	1.5
	Infectious diseases	7	0.6
	Birth Defects and disabilities	6	0.5
	Rare diseases	5	0.4
	Chemotherapy	6	0.5
**Duration of the disease (Year)**	1–10	1061	45.92
	11–20	104	41.76
	> = 20	61	10.4
**Experience of using telemedicine**	Yes	171	13.9
	No	1055	86.1

In the diseases section, each patient could choose more than one disease

### Data analysis

Demographic and clinical information of patients (first and second part) was analyzed by calculating frequency and percentage. Also, in order to analyze part three of the questionnaire, descriptive statistics (mean, standard deviation) were used. Chi-square test was used to check the significant difference between choosing telemedicine and in-person visit based on demographic and clinical characteristics of the participants. All these analyses were done in SPSS 23.

In order to analyze the data related to the open question, we adopted an inductive approach, employing Graneheim and Lundman’s qualitative content analysis methods to link the data analysis to the specific research questions [33, 34]. All the answers of 1026 patients were typed in a word file and imported into MAXQDA 18 software for qualitative analysis. Each individual response underwent a meticulous assessment, from which the core units of significance were extrapolated through the conventional content analysis approach, these were then encoded under the supervision of the three authors. The analysis delved into words, sentences, and sentence fragments. At the word level, nuances in meaning, connotations, and associations were identified. Moving on to sentences, the study revealed expression of ideas, employed syntactic structures, and overall coherence. Fragments of sentences were also scrutinized, capturing nuances within incomplete expressions.

To ensure the robustness of the analysis, a multi-step validation process was enacted. Initially, the extracted codes underwent a thorough review, leading to the categorization of codes into primary themes and corresponding sub-themes, guided by shared semantic contexts. The subsequent stage entailed a reevaluation of the responses, codes, and thematic structures by a panel of three accomplished medical informatics experts and health information management.

It should be noted that, these experts possess a robust background in qualitative research methods, gained from diverse projects involving content analysis, encompassing coding, theme development, and pattern recognition in textual data. Their experience stems from coursework, mentorship, and prior research. With extensive involvement in various qualitative studies and a strong publication record, they showcase skill in thematic analysis, coding coherence, and data interpretation.

### Ethical considerations

To conduct this study, ethics approval (IR.KMU.REC.1401.390) was obtained from the Research Ethics Committee of Kerman University of Medical Sciences. Prior to participation in the study, informed consent was obtained from the patients.

## Results

Out of the total participants, 922 (75.2%) were women. The largest age group was between 18 and 27 years old. The majority of participants resided in metropolitan areas (1105 patients). Furthermore, a significant number of participants reported having oral and dental diseases (359 patients). A total of 171 participants (13.9%) had prior experience using telemedicine (Table 1).

Among the 1026 surveyed patients, 856 (71%) favored in-person visits, while 361 (29%) preferred telemedicine (Table 2). The Chi-square test revealed a significant difference in preference percentages, with in-person visits being more popular (Chi-Square = 91.58; *p* < 0.0001). Regardless of gender and location, most participants leaned towards in-person visits. For individuals with a PhD, those with specific conditions (skin/hair, musculoskeletal, disabilities), and those experienced with telemedicine, the preference skewed towards telemedicine visits (*p* < 0.0001).

**Table 2:** Selection of telemedicine or in-person visit based on patients’ demographic characteristics

**Variables**		**Total frequency**	**In-person visit**	**Telemedicine visit**	***P*** **-value**
			**Frequency (%)**	**Frequency (%)**	
**Sex**	Male	304	207 (68.1)	97 (31.9)	< 0.0001
	Female	922	658 (71.4)	264 (28.6)	
**Age**	18–27	661	474 (71.7)	187 (28.3)	0.787
	28–37	370	253 (68.4)	117 (31.6)	
	38–47	159	114 (71.7)	45 (28.3)	
	48–57	29	19 (65.5)	10 (34.5)	
	> 57	7	5(71.4)	2 (28.6)	
**Education level**	Diploma	441	324 (73.5)	117 (26.5)	< 0.0001
	Associate degree	103	70 (68.0)	33 (32.0)	
	Bachelor	480	337 (70.2)	143 (29.8)	
	Master	156	102 (65.4)	54 (34.6)	
	PhD	46	14 (30.4)	32 (69.6)	
**Residence Type**	Metropolitan areas	1105	772 (69.9)	333 (30.1)	< 0.0001
	Remote and rural areas	121	93 (76.9)	28 (23.1)	
**Disease type**	Oral and dental diseases	359	200 (55.71)	159 (44.28)	< 0.0001
	Eye diseases	291	200 (68.72)	91 (22.27)	
	Skin and hair diseases	281	130 (46.26)	151 (53.73)	
	Digestive diseases	254	154 (60.62)	100 (39.37)	
	Otorhinolaryngology diseases	190	110 (57.89)	80 (42.10)	
	Gynecological diseases	140	109 (77.85)	31 (22.14)	
	Endocrine diseases	121	115 (95.04)	6 (4.95)	
	Psychological disorders	95	83 (87.36)	12 (12.63)	
	Musculoskeletal diseases	101	45 (44.55)	56 (55.44)	
	Cardiovascular diseases	85	65 (76.47)	20 (23.52)	
	Respiratory diseases	61	32 (52.45)	29 (47.54)	
	Cancers	10	10(100)	0(0)	
	Rheumatology diseases	28	19 (67.85)	9 (32.14)	
	Kidneys and urinary diseases	73	50 (68.49)	13 (31.50)	
	Genetic disorders	18	12 (66.66)	6 (33.33)	
	Infectious diseases	7	5 (71.42)	2 (28.51)	
	Birth Defects and disabilities	6	2 (33.33)	4 (66.66)	
	Rare diseases	5	4 (80)	1 (20)	
	Chemotherapy	6	6 (100)	0 (0)	
**Duration of the disease**	1–10	1061	746 (70.3)	315 (29.7)	< 0.0001
	11–20	104	77 (74.0)	27 (26.0)	
	> = 20	61	19 (31.1)	42 (68.9)	
**Experience of using telemedicine**	Yes	171	70 (40.9)	101 (59.1)	< 0.0001
	No	1055	746 (72.4)	291 (27.6)	

Considering participant consensus or mean scores, three primary telemedicine benefits prominently emerged: avoiding infectious diseases, saving cost, and eliminating and overcoming geographical distance barriers. Quick diagnosis of the disease, better diagnosis of the disease and reduction of medical errors also received the lowest mean scores (Table 3).

**Table 3:** Advantages of using telemedicine versus in-person visit

**Row**	**Advantages**	**In-person visit**	**Telemedicine visit**	***P*** **-value**
		**Mean (SD)**	**Mean (SD)**	
1.	Avoiding infectious diseases	3.89(1.02)	4.30(0.83)	< 0.0001
2.	Saving costs	3.52(0.98)	4.70(0.87)	< 0.0001
3.	Eliminate and overcome geographical distance barriers	3.76(0.98)	4.24(0.78)	0/036
4.	Spending less time to receive medical services	3.58(1.01)	4.12(0.83)	< 0.0001
5.	Schedule the next visit	3.38(1.15)	3.99(0.76)	< 0.0001
6.	Easy electronic collection and storage of medical data and information	3.32(1.02)	3.91(0.85)	0/045
7.	Convenient sharing of knowledge and information between therapists when faced with a rare disease	3.27(1.03)	3.88(0.94)	< 0.0001
8.	Reducing patient anxiety and stress	3.19(1.09)	4.07(0.80)	0/014
9.	Less embarrassment and shame of the patient in providing information to the therapist	3.18(1.53)	4.02(0.94)	0/011
10.	Easy to pay medical bills	3.17(1.02)	3.89(0.88)	< 0.0001
11.	Better patient-therapist interaction	2.77(1. 04)	3.89(0.88)	< 0.0001
12.	Maintaining confidentiality and privacy of information	3.18(1.15)	3.90(0.92)	< 0.0001
13.	Increasing the patient’s self-confidence in performing treatment processes	3.09(1.05)	3.92(0.87)	0/043
14.	Easily perform self-care and self-management processes	2.97(0. 98)	3.88(0.83)	< 0.0001
15.	Easy exchange of information between the patient and the therapist	2.90(1. 07)	3.97(0.88)	0/049
16.	Easy reporting of drug and treatment side effects to the physician	2.37(0. 87)	3.83(0.84)	< 0.0001
17.	Easy presentation of the patient’s treatment history to the therapist	2.87(1. 05)	3.93(0.85)	< 0.0001
18.	Better presentation of the patient’s current history to the therapist	2.84(1. 07)	3.94(0.86)	0/03
19.	More patient peace of mind for treatment	2.93(1. 13)	3.81(0. 87)	0/041
20.	Better and easier follow-up of treatment instructions	2.87(1. 06)	3.77(0. 87)	0/042
21.	Better and easier use of health insurance	2.92(1. 05)	3.59(0.96)	0/021
22.	Greater honesty of the patient in providing information to the therapist	2.81(1.10)	3.75(1.02)	< 0.0001
23.	Getting better treatment advice	2.85(1.10)	3.79(0.88)	0/038
24.	Improving lifestyle	2.65(1.13)	3.73(0.82)	0/02
25.	Easy follow-up of medical malpractices	2.85(1. 13)	3.58(0.99)	0/049
26.	Giving more time to the therapist to treat the patient	2.73(1.00)	3.65(1.01)	< 0.0001
27.	Better interpretation of laboratory tests	2.68(1. 09)	3.65(0.90)	< 0.0001
28.	Professional commitment of the therapist to treat the patient	2.66(1. 03)	3.54(0.96)	< 0.0001
29.	Making better and more accurate treatment decisions by the therapist	2.45(0.95)	3.49(0.93)	< 0.0001
30.	Receive quality medical services	2.47(0.93)	3.49(0.94)	0/01
31.	Greater medication adherence	2.47(0.89)	3.35(0.97)	0/021
32.	Better and more accurate medicine prescription	2.42(0.91)	3.44(0.94)	0/039
33.	Faster recovery of the patient	2.37(0.87)	3.33(0.95)	0/034
34.	Reduction of medical errors	2.33(1.01)	3.31(1.00)	0/033
35.	Better diagnosis of the disease	2.19(0.94)	3.25(1.08)	< 0.0001
36.	Quick diagnosis of the disease	2.22(0.93)	3.20(1.05)	< 0.0001

### Reasons for choosing an in-person visit or telemedicine

A total of 401 reasons for in-person or telemedicine visits were identified. Through an iterative process, similar items were aggregated, resulting in the formation of 183 main reasons (different reasons for choosing either telemedicine (108 reasons for telemedicine and 75 reasons for in-person visits).

#### Reasons for choosing an in-person visits 

A total of eighty-two reasons for opting for an in-person visit were categorized into ten main themes: physical examination, disease diagnosis, treatment, prescription and drug consumption, face-to-face communication with the physician, psychological aspects, insurances and reimbursements, information technology infrastructure, security and privacy, and laboratory tests. Among the themes, treatment had the most cited sub-themes. “More accurate diagnosis of the disease”, “More accurate and better examination of the patient by the physician” and “More accurate and better treatment of the disease” were the primary reasons for selecting an in-person visit. Table 4 displays the rationales behind patients’ choice of in-person visits.

**Table 4:** Reasons for choosing an in-person visit

**Main themes**	**Sub-themes**	**Number of quotes**
**Physical examination**	Detailed and complete physical examination of the patient	217
	High quality examination tools in in-person visit	10
	More accurate assessment of the patient	8
	Reduction of medical errors by physical examination	5
	Accurate decision-making by the physicians	1
	More physician focus on the patient’s words	1
	Physician paying more attention to the patient	1
	Spending more time for the patient’s examination by the physician	1
**Disease diagnosis**	More accurate diagnosis of the disease	301
	Reducing misdiagnoses	68
	More accuracy of the physician in the examination	30
	Faster diagnosis of the disease by the physician	6
	Providing a better clinical history by the patient	5
	Lack of use of telemedicine in diagnosing some diseases	3
	More accurate understanding of the symptoms by the physician	2
	Diagnosis of disease severity	1
	Making treatment decisions faster	1
	Reducing misconceptions	1
	Provide more accurate, clearer and more honest explanations about the disease and its symptoms by the patient	1
	Easy interpretation and analysis of the disease	1
	Determining the exact location of the pain by the patient	1
**Treatment**	More accurate and better treatment of the disease	186
	More confidence and trust in-person visits	105
	Faster treatment	35
	More adherence of the patient to the treatment	18
	More commitment of the physician to treat the patients	5
	Better and more complete exchange of patient information with the physician	4
	Reducing medical errors and the risks of treatment	3
	More motivation of the physician to treat the patients	3
	Better understanding of how to perform post-treatment processes	1
	Easier remembering of previous treatments done for the patient	1
	Receiving medical services more easily from the nurses	1
	Reducing treatment problems for the patients	1
	High quality of treatment in in-person visits	1
**Prescription and Medication**	Better prescription of medicines	43
	More accuracy of physician in prescribing medicines and fewer errors in prescriptions	2
	Regular use of medicines	1
	Fewer drug side effects	1
**Face-to-face communication with the physician**	Direct, visual, comfortable and better communication between the patient and the physician	84
	Physician paying more attention to the patient	33
	Patient’s clear response to the doctor’s questions	10
	Closer communication between the patient and the physician	20
	Providing better quality services	12
	Providing a better clinical history by patient	3
	Comfortable interaction of children with the physician	2
	Not neglecting patient-provided clinical information to the physician	1
	Better guidance of the patient in carrying out treatment processes	1
**Psychological aspects**	Feeling better and more confident about the quick treatment of the disease	12
	More peace of mind for the patient during the treatment	6
	Creating a positive feeling to continue further treatment	5
	Patient’s mastery of speaking to provide a history to the physician	1
	More understanding of the patient by the physician	1
	Traditional and religious view of accepting in-person visits	
	Increasing patients’ self-confidence	1
	Habit of visiting the clinic in person	1
**Insurance and reimbursement**	Being covered by insurance for drugs for certain diseases	1
	Use of insurance	1
**Information technology infrastructure**	Lack of computer infrastructure and strong network for telemedicine	21
	No need for computer and digital knowledge and literacy	17
	Constant Internet outages	1
	Low bandwidth	11
	Lack of culture to use technologies	11
	No need for computers and other powerful communication equipment such as mobile phones	4
	Slow internet speed	1
	Lack of experience in using telemedicine	1
**Security and privacy**	Maintaining patient privacy	5
	Maintaining confidentiality of information	3
	Fearing of easy disclosure of information in telemedicine	2
	Maintaining information security	1
	Lack of clear rules in the field of electronic disclosure of patient information	1
	Cyber fraud	1
	Hacking and unauthorized access to medical record information	1
	Fraud and abuse of patient data	1
**Laboratory tests**	Necessity of conducting clinical tests in person	1
	Better interpretation of the results of clinical tests	1

#### Physical examination

The patients believed that an in-person visit allows for a thorough and comprehensive physical examination, precise and accurate decision-making by physicians, increased physician attention to the patients, lowered risk of medical errors, improved patient assessment accuracy, and a more comprehensive understanding of the individual’s condition.

“I agree with the in-person visit because the examination is physical, and the physician can provide a more accurate diagnosis.” (Participant 200)

“I believe a more comprehensive examination is possible in-person, aiding the physician’s accurate decision-making.” (Participant 305)

#### Disease diagnosis

Correct and timely diagnosis was another reason for patients choosing an in-person visit.

“I dislike telemedicine as the physician’s accurate diagnosis seems possible only in-person.” (Participant 105).

#### Treatment

The most frequently cited reasons for choosing in-person visits were related to treatment. Patients chose in-person visits for several reasons, such as: more accurate and better treatment of the disease, more confidence and trust in-person visits, faster treatment, more adherence of the patient to the treatment, more commitment of the physician to treat the patients, better and more complete exchange of patient information with the physician, reducing medical errors and the risks of treatment, more motivation of the physician to treat the patients, better understanding of how to perform post-treatment processes, easier remembering of previous treatments done for the patient, receiving medical services more easily from the nurses, reducing treatment problems for patients, and high quality of treatment in in-person visits.

*“In my view, in-person *visits* are unmatched by telemedicine. I strongly believe they offer superior, more accurate, and effective treatment.” (Participant 505)*

*“I have more faith in my treatment process during in-person visits, expecting quicker and more effective care.” (Participant 321)*

*“Unlike telemedicine, I sense that the physician’s dedication to treating the patient is *considerably* greater during an in-person visit. This is why I never opt for telemedicine.” (Participant 71).*

#### Prescription and medication

Patients pointed out that in-person visits can lead to better prescription of medicines, regular use of medicines, more accuracy of physician in prescribing medicines and fewer errors in prescriptions and fewer drug side effects.

*“In the *clinic*, when the patient visits in person, the physician can directly assess test results and the patient’s condition. This leads to more precise medication prescriptions.” (Participant 41)*

*“During in-person visits, I feel the physician allocates more time to me, resulting in better medication prescriptions that I consistently follow.” (Participant 320)*

*“The in-*person* physician’s cautious medication prescriptions lead to fewer drug side effects.” (Participant 901)*

#### Face-to-face communication with the physician

In-person visits were underscored by patients as they enabled direct face-to-face communication with the physician, leading to heightened physician attentiveness, improved clinical history collection, and a more focused, comfortable interaction between patients and physicians. This approach also resulted in clearer patient responses, stronger patient-physician relationships, enhanced service quality, effective transmission of clinical information, ease of interaction for children, and improved guidance throughout treatment processes.

*“During in-person visits, I’m certain the physician attentively listens to what I say.” (*Participant* 12)*

*“I believe that more effective treatment occurs when there’s direct visual and verbal communication between the physician and the patient.” (Participant 1002)*

*“I believe direct patient-physician interaction in-person boosts physician accuracy and focus more than telemedicine. That’s why I exclusively visit clinics for treatment. For this reason, I choose to only seek treatment by physically visiting clinics.” (Participant 679)*

#### Psychological aspects

In this category, creating a positive feeling to continue further treatment, the patient’s mastery of speaking to provide a history to the physician, feeling better and more confident about the quick treatment of the disease, more peace of mind for the patient during the treatment, more understanding of the patient by the physician, traditional and religious view, increasing self-confidence and the habit of visiting the office in person were the most important reasons of patients to choose an in-person visit.

*“During an in-person office visit, I feel more inclined to pursue additional treatment.” (Participant 718)*

*“Personally, seeing a physician in person brings me a sense of peace that outweighs a faster recovery, as I trust their in-depth understanding of my condition leads to a more accurate diagnosis.” (Participant 347)*

*“I will have more self-confidence because I talk face-to-face with the physician during the in-person visit.” (Participant 417)*

#### Insurance and reimbursement

Patients believed that in in-person visits, drugs related to certain diseases are covered by insurance and it is possible to use insurance easily.

*“I have MS and require specific medications. Affording these drugs without insurance is unfeasible due to my financial situation, and I’m unsure if my insurance covers telemedicine visits and prescriptions.” (Participant 197)*

*“In-person visits offer more accurate clinical examinations, improved patient care from physicians, and notably, the ability for patients to utilize insurance.“ (Participant 521)*

#### Information technology infrastructure

Other reasons for choosing an in-person visit were: lack of suitable computer and networking infrastructure for telemedicine, slow Internet speed, frequent internet outages, low bandwidth, no need for computer and digital knowledge and literacy, no need for computers and other communication equipment such as mobile phones, lack of culture to use technologies and lack of experience in using telemedicine.

*“Telemedicine infrastructure should be built in our country first, then we should use telemedicine, I think there is still no strong infrastructure.” (Participant 359)*

*“I live in a village and I am reluctant to use telemedicine due to the slow internet speed in my area.” (Participant 344)*

*“Due to my parents’ elderly status, using telemedicine is challenging for them as they lack computer and internet skills. Hence, in-person visits are more suitable for them.” (Participant 81)*

*“During an in-person visit, there’s no requirement to use devices like laptops or mobile phones for communication with the physician. Additionally, my economic situation does not allow me to afford such tools.” (Participant 651)*

*“I believe that a culture of using telemedicine has not yet fully developed, and if we intend to adopt it, we are likely to encounter numerous challenges.” (Participant 25)*

#### Security and privacy

Challenges related to the fear of easy disclosure of information in telemedicine, maintaining patient privacy, maintaining information security, maintaining confidentiality of information, lack of clear rules in the field of electronic disclosure of patient information, cyber fraud, hacking and unauthorized access to medical record information, fraud and abuse of patient data were other reasons to the choice of in-person visits over telemedicine.

*“Due to my unique medical condition, I choose not to use telemedicine as I am constantly concerned about information disclosure. I believe that sharing information is much more straightforward in telemedicine compared to an in-person visit.” (Participant 219)*

*“In my view, a telemedicine system might jeopardize patients’ privacy by potentially sharing their sensitive data with third parties for personal financial gains.” (Participant 44)*

*“In my opinion, the prevalence of cyber fraudsters who can readily exploit patients’ data is a significant concern, which is why I choose not to use telemedicine at all.” (Participant 49)*

*“In my opinion, there is a lot of possibility for fraud and abuse of patient data in telemedicine, which makes people less likely to go to this technology. Therefore, they prefer to use in-person visits.” (Participant 953)*

#### Laboratory tests

The necessity of conducting laboratory tests in person and better interpretation of the results of these tests were the reasons for choosing in-person visits.

*“I believe that tests like mammography, pap smear, ultrasound, and eye pressure testing for glaucoma should be conducted in person.” (Participant 852)*

##### Reasons for choosing telemedicine

A total of 108 reasons for choosing telemedicine to facilitate treatment processes were categorized into 20 main themes: patient and provider safety, physical examination, diagnosis, treatment, medication prescription, access to physicians, cost management, time management, geographical distances, psychological aspects, electronic registration and data management, insurance and reimbursement, healthy environment, interaction and communication, information technology, security, privacy and legal issues, laboratory tests, remote monitoring, and admission and hospitalization. Like the in-person visit in telemedicine, the theme of treatment had the most sub-themes. The most important reasons for choosing telemedicine were “avoiding infectious diseases and preventing their further spread”, “reducing treatment costs” and “saving time” (Table 5).

**Table 5:** Reasons for choosing telemedicine

**Main themes**	**Sub-themes**	**Number of quotes**
**Patient and provider safety**	Reduce the incidence of infectious diseases and prevent their further spread	296
	Protect service providers against infectious diseases	2
	Initial assessment and triage of patients with symptoms of infectious diseases	1
	Increase patient safety	1
	Vaccination at national level	1
**Physical examination**	Access to patients’ history	1
	Spending more time and time of the physician for the patient’s examination	11
	Not forgetting the information that can be provided in describing the history by the patient	2
	More honesty of the patient in providing information	1
**Diagnosis**	Physician’s greater accuracy in diagnosing the disease and avoiding their mistakes during the diagnosis process	4
	Access to the patient’s clinical history	1
	Physician’s access to reliable information	1
	Reduction of life and financial complications	1
**Treatment**	Easier, better and faster treatment	21
	Improvement of patient participation in treatment processes	4
	Easy finding of answers to questions	3
	Visiting and treating a larger number of patients	3
	Managing and treating the elderly comfortably	3
	Helping the patient in emergency situations	2
	No need for in-person visits	2
	Easy management and control of chronic diseases	2
	Increasing individual independence	2
	Allocating more time by the physician to the patient	1
	Recording all treatment steps	1
	Reviewing and accessing the patient’s electronic record	1
	More energy of physicians to treat patients	1
	More responsibility of physicians	1
	Training on how to treatment processes for patients	1
	Providing fast care services to patients	1
	Improving self-care processes	1
	Compensating for the lack of physicians in treatment centers	1
	More accurate treatment recommendations	1
	Spending more physician time to follow up the patient’s treatment processes	1
**Medication prescription**	Better monitoring of patients’ health status by physician to prescribe medicine	1
	Better prescription of medicine for individuals with chronic diseases	1
**Access to physicians**	Access to wider range of specialties	45
	Quick and easy access to physicians at any time and from any place	11
**Cost management**	Reducing treatment costs	210
	Reduce consumption of personal protective equipment	1
	Reduce fuel consumption	1
	Simple payment of costs	1
	Reduce costs related to office and paper processes	1
	Reduce accounting errors	1
	Increase the income of physicians and health organizations	1
**Time management**	Saving time	181
	Control and manage time better	2
	Reduce patient waiting time	2
**Geographical distances**	Eliminating geographical distances	9
	Reducing the need for additional trips and the possibility of traveling to any medical center anywhere in the world	5
	Receiving emergency care services quickly	3
	Expanding the geographic range to receive healthcare services	2
**Psychological aspects**	Comfortable interaction of shy and isolated individuals with the therapist	19
	Reducing tension and stress	15
	More peace of mind for patients in treatment	4
	Increasing patients’ self-confidence	2
	Increasing patients’ motivation to continuing his treatment	2
	Reducing social stigma	1
	Reducing fear and stress caused by reducing time	1
	Less stress of service providers	1
	Faster recovery of patients with mental disorders	1
**Electronic registration and management of patients’ information**	Saving the electronic information of the patients	3
	Access to clinical records of patients at any time and any place	2
	Quickly retrieve the information	1
	Reducing the use of paper	1
	Don’t spend a lot of time by physicians to obtain the required information	1
	Save audio and video information	1
	Access to up-to-date information by physicians	1
	Easy sharing of patient information with other physicians	1
	Preservation of information against natural events	1
	Transfer of patient information to medical centers	1
**Insurance and reimbursements**	Easy receipt of bills from insurance companies	1
	Providing logical evidence against insurance companies’ claims	1
**Healthy environment**	Reducing urban traffic	5
	Not destroying green spaces	2
	Increasing the physical and mental health of patients by eliminating noise pollution	2
	Reducing pollution	1
**Interaction and communication**	Reducing the stress and anxiety of the patients	5
	Better and easier interaction with the therapist	3
	Establishing private communication between the patient and the physician	2
	Peer-to-peer support	1
	Consulting physicians with each other	1
**Information technology**	Creating a backup of medical records	2
	Reduce repetitive processes	1
	Training self-care processes to patients	1
	Allowing to patients to access their medical information	1
**Security, privacy and legal issues**	Determination of authorized persons to use patient information	1
	Easy identification of persons who disclose information	1
	Determination of access levels to medical records	1
	Prevention of information losing	1
	Reduction of unauthorized use of information	1
	Easy follow-up of legal issues	1
**Laboratory tests**	Storage and quick access to laboratory test results by patients	1
	Quick sending of laboratory test results to therapists	1
	Better interpretation of test results	1
	Reduction of in-person visits of patients to medical centers to perform laboratory tests	1
**Remote monitoring**	Vital sign monitoring for control and management of patients	5
	Easy drug withdrawal for addicts	2
	Reducing the exacerbation of the disease	1
	Monitoring and controlling the health status of veterans	1
	Providing easy services to disabled individuals	1
	Easy care of babies and children	2
	Providing telerehabilitation services to patients	2
	Use of monitoring tools and sensors to monitor the condition of patients and save their lives	1
**Admission and hospitalization**	Help individuals living in villages to easily visiting	17
	Easily determine appointment times	7
	reducing the rate of hospital admissions and readmissions	4
	reducing the crowding of medical centers and hospitals	4
	Increasing the number of patients visited	1

#### Patient and provider safety

Patients believed that telemedicine can help reduce the incidence of infectious diseases and prevent their further spread, protect service providers against infectious diseases, initial assessment and triage of patients with symptoms of infectious diseases, increase patient safety, and vaccination at national level.

*“Currently, due to infectious diseases such as COVID-19, I prefer not to have an in-person visit and instead opt for telemedicine.” (Participant 230)*

*“I work in a hospital and believe that the primary advantage of telemedicine is its ability to minimize direct contact between providers and patients.” (Participant 765)*

*“A great feature of telemedicine is its ability to perform initial assessments and triage for patients with symptoms of infectious diseases like COVID-19. This allows for prompt identification and guidance towards suitable treatment.” (Participant 413)*

*“Now that COVID-19 has spread, I think one of the most important applications of telemedicine is national vaccination. Telemedicine can be used to encourage people to get vaccinated as soon as possible through video conferences or text reminder messages.” (Participant 310)*

#### Physical examination

Patients have noted that telemedicine offers easier history-taking, allowing more time for physicians to examine patients. This approach ensures that patients can provide a comprehensive medical history and share information more honestly.

*“I personally believe that I can more easily explain my medical history and concerns to the therapist through telemedicine.” (Participant 485)*

*“I have had the experience of using telemedicine once or twice. In my opinion, during telemedicine appointments, physicians devote more time to examining patients.” (Participant 1012)*

*“During in-person visits, long waits and rushed consultations due to high patient volume can stress patients and lead to missed important illness details.” (Participant 963)*

*“When using telemedicine, I find that I can provide the physician with a more honest presentation of my clinical information and medical history.” (Participant 1125)*

#### Diagnosis

Patients also opt for telemedicine due to factors such as the physician’s enhanced accuracy in diagnosing diseases, minimized diagnostic errors, access to the patient’s complete clinical history, reliable information accessibility for physicians, decreased clinical complications, and cost savings.

*“I find telemedicine consultations to be better because I believe physicians are more accurate in diagnosing my condition.” (Participant 842)*

*“In my view, telemedicine leads to better diagnosis and management of diseases, resulting in reduced clinical and financial burdens for the patient.” (Participant 514)*

#### Treatment

Patients prefer telemedicine due to reasons like streamlined treatment with improved quality and speed, convenient access to electronic records, and comprehensive recording of treatment steps. Additionally, they believed that telemedicine offers quick emergency responses, efficient chronic disease management, and enhanced patient engagement Moreover, telemedicine addresses physician shortages, facilitates self-care, and ensures precise treatment recommendations, all while increasing physician involvement in monitoring treatment progress.

*“I’m very fond of telemedicine because I believe it makes my treatment process easier and faster.” (Participant 574)*

*“I believe that in emergency situations, physicians can manage and control patients much more quickly online.” (Participant 1019)*

*For individuals with a chronic disease, telemedicine is highly beneficial as it enables them to enhance their lifestyle and manage their condition through convenient and easy communication with their physician.” (Participant 1001)*

*“As a disabled person still living with my family, when I fall ill, I need to visit the medical center accompanied by a family member. However, with telemedicine, I can readily undergo treatment without relying on anyone.” (Participant 219).*

*“Telemedicine enables me to easily manage self-care processes, treatments, and take better care of myself.” (Participant 917)*

#### Medication prescription

Patients believed that telemedicine could facilitate improved health status monitoring by physicians for prescribing appropriate medicines, particularly benefiting individuals with chronic diseases.

*“From my perspective, telemedicine holds value in prescribing medication for individuals dealing with chronic conditions. It facilitates convenient communication with physicians to obtain personalized prescriptions. Furthermore, it proves useful in addressing drug side effects through telemedicine consultations, providing guidance on proper usage and the management of emerging effects.” (Participant 444).*

#### Access to physicians

Patients believe that telemedicine provides access to a wider range of specialties and enables quick and easy access to physicians at any time and from any place.

*“When I was diagnosed with Covid-19, I could easily communicate with my physician brother who lives in Canada and receive medical advice.” (Participant 1009)*

*“I live in a village and can’t go to the hospital or the hemophilia treatment center in the city every day for my treatment. With telemedicine, I can easily communicate with my physician anytime and anywhere.” (Participant 770)*

#### Cost management

Patients note that telemedicine can lower treatment expenses, reduce personal protective equipment usage, decrease fuel consumption, simplify payments, minimize office-related costs, cut paper-related expenses, decrease accounting errors, and boost healthcare providers’ and organizations’ income.

*“Due to my limited economic situation, I prefer using telemedicine as I can’t afford to visit medical centers every day.” (Participant 1209)*

*“Another advantage of telemedicine is the reduction in fuel consumption, such as gasoline, as individuals do not need to visit the clinic in person.” (Participant 1204)*

*“I endorse telemedicine for its ability to markedly cut costs related to office and paper processes, postage, printing, and paper-based information storage.” (Participant 409)*

#### Time management

Patients have indicated that telemedicine can save time, enable better time management, and decrease patient waiting times.

*Due to the widespread Covid-19 situation, minimizing medical center referrals is essential, saving patients from lengthy wait times. (Participant 883)*

*“I am confident that telemedicine allows me to better manage my time and plan my daily activities more effectively.” (Participant 706)*

#### Geographical distances

According to patients’ perspective, telemedicine can eliminate geographical barriers, minimize the need for extra trips, provide access to medical centers globally, offer prompt emergency care services, and expand the reach to access healthcare services across larger geographic areas.

*“Telemedicine not only enables individuals with mental health conditions, who are particularly susceptible to self-harm and suicide, to consult a therapist or psychiatrist promptly day or night, but it also seamlessly extends healthcare services’ geographic reach to anywhere in the world.” (Participant 174)*

*“Telemedicine can effortlessly extend the geographic reach of healthcare services to anywhere in the world.” (Participant 967)*

#### Psychological aspects

Patients choose telemedicine for psychological benefits, experiencing reduced fear, stress, and anxiety. This leads to greater peace of mind during treatment, lowers stress for providers, reduces social stigma, boosts patient self-confidence, enhances motivation to continue treatment, and accelerates recovery for mental health patients. Additionally, telemedicine offers a comfortable platform for shy and isolated individuals to interact with therapists.

*“I favor telemedicine for its convenience and peace of mind during treatment, offering me increased confidence and easy communication with the physician.” (Participant 97)*

*“ I believe telemedicine’s key benefit is seen in socially stigmatized conditions like HIV and addiction, where patients feel at ease with physicians, enabling open sharing of information.” (Participant 54).*

*“I believe telemedicine not only fosters improved physician and therapist ethics, encouraging patient commitment to treatment, but also aids isolated and shy individuals by alleviating shyness and creating a comfortable space for sharing information with therapists.” (Participant 710).*

#### Electronic registration and management of patients’ information

Patients cite advantages like preserving electronic records, fast data retrieval, cutting paper use, minimizing physician data-gathering time, storing audio/video data, informed decisions using clinical records, accessing current information, sharing patient data easily among peers, and safeguarding data from natural events.

*“Telemedicine facilitates electronic exchange of patient information between patients and physicians, enabling the electronic storage and management of clinical data and information, consequently minimizing the requirement for paper usage.” (Participant 7)*

*“In my view, another significant advantage of telemedicine is its ability to effectively safeguard patient information against natural events like floods, earthquakes, and more.” (Participant 4)*

#### Insurance and reimbursements

Patients noted that telemedicine simplifies insurance reimbursement for healthcare providers, irrespective of their location, and enables them to furnish official care evidence for insurance claims.

*“In my opinion, telemedicine simplifies the process of providing legal evidence for insurance claims compared to in-person visits.” (Participant 367)*

*“ I believe that in telemedicine, insurance companies enable doctors and other therapists to bill for the healthcare services they offer, regardless of the patient’s or provider’s location.” (Participant 19)*

#### Healthy environment

In the theme of healthy environment, the patients emphasized on reasons such as reducing pollution, not destroying green spaces, reducing urban traffic and increasing the physical and mental health of patients by eliminating noise pollution.

*“I don’t like in-person visits because of my asthma. In polluted environments, my disease worsens, with in-person visits, travel between or within cities increases and the environment becomes more polluted. (Participant 10)”*

*“I love nature and green spaces. My top reason for choosing telemedicine is to lower pollution by minimizing in-person medical visits, which in turn helps preserve the environment.” (Participant 38).*

*“I appreciate telemedicine as it relieves me from city traffic fatigue. It helps reduce urban traffic congestion.” (Participant 1012)*

*“I believe car-related noise pollution is a major issue in urban areas, posing a threat to mental health, particularly for hospital patients. Through telemedicine, additional trips to hospitals and medical centers can be reduced and noise pollution can be prevented.” (Participant 1068)*

#### Interaction and communications

Patients value telemedicine for enhancing therapist interactions, enabling private communication, facilitating peer support, enabling physician consultations, and reducing stress/anxiety.

*“Telemedicine not only enables convenient communication between patients and physicians, preventing unnecessary time waste, but also alleviates the stress of in-person medical center visits, resulting in more relaxed interactions.” (Participant 988)*

*“ I get stressed when I want to go to medical centers in person and communicate with my physician, while with telemedicine I am not stressed at all.” (Participant 45)*

#### Information technology

Some patients have mentioned that telemedicine aids in training patients for self-care processes, decreasing repetitive tasks, generating medical record backups, and enabling patient access to their medical information.

*“Another advantage of telemedicine is its ease in delivering self-care training to patients through multimedia technologies following their treatment. (Participant 1)”*

*“Through telemedicine, numerous repetitive processes can be minimized, such as multiple visits to medical centers for medicine renewal. New medicines can be prescribed each time based on the patient’s current condition. (Participant 13)”*

#### Security, privacy and legal issues

Patients opt for telemedicine due to reasons such as authorizing information access, identifying information-disclosing individuals, setting medical record access levels, preventing data loss, reducing unauthorized information use, and facilitating legal issue follow-ups.

*“I highly value my medical privacy and vehemently oppose unauthorized use of my information. Telemedicine can significantly reduce such unauthorized access and enable patients to determine who will have access to their information.” (Participant 27)*

*“When I visited medical centers in person several times, some of my information was lost, such as my test sheets. While in telemedicine, the possibility of losing patient information, especially the results of laboratory tests, is very low.” (Participant 209)*

*“Due to the advanced technologies used in telemedicine, it becomes easy to identify individuals disclosing patient information, and it also simplifies addressing legal matters for both patients and physicians.” (Participant 22)*

#### Laboratory tests

Patients emphasized their choice of telemedicine due to streamlined storage and quick access to lab results, rapid transmission to therapists, enhanced result interpretation, and reduced in-person visits for tests.

*“Telemedicine offers multiple advantages. It allows me to upload and store my lab test results on websites for easy access when needed by me or my physicians. Additionally, it reduces costs and distances while enabling swift transmission of laboratory test results to physicians.” (Participant 666).*

*“I believe that in telemedicine, physicians interpret the results of lab tests much more effectively. Unlike in-person visits, they aren’t dealing with a large population of patients, and medical centers are not as crowded.” (Participant 946).*

#### Remote monitoring

Patients recognized the telemedicine potential of remote monitoring, which involves using tools and sensors to oversee conditions, leading to life-saving measures. Moreover, this approach prevents disease exacerbation, enables health control, aids disabled individuals, monitors vital signs, assists in drug withdrawal, cares for infants and children, and provides telerehabilitation.

*“Telemedicine utilizes monitoring tools and sensors to track patients’ conditions and relay alerts to physicians, notifying them of health deterioration and emergencies.”(Participant 57)*

*“I am a chemical veteran, and I have to visit the physician’s office every week. Telemedicine could enable remote monitoring, alleviating the need for frequent visits.” (Participant 70)*

*“We have a disabled brother in the family who cannot speak or walk properly. Every month, we have to take him to medical centers for doctor’s monitoring. Telemedicine seems promising for providing medical care to disabled patients.” (Participant 1014)*

*“Through telemedicine, physicians can monitor patients in the ICU and monitor their vital signs, such as pulse rhythm, blood pressure, glucose levels, and more.” (Participant 1009)*

*“In my opinion, by using telemedicine, addicts can better quit their addiction, because physicians can monitor and control them more.” (Participant 1072)*

#### Admission and hospitalization

As per patients’ opinions, telemedicine is regarded as advantageous for individuals in rural areas. It provides convenient visits, streamlined appointment scheduling, decreased hospital admissions/readmissions, less crowded medical facilities, and enhanced patient capacity.

*“I live in the village, and it is very difficult for me to travel to the city every week for my physician to visit me, but with telemedicine it will be easier for me to be visited.” (Participant 104)*

*“I dislike crowded medical centers; I believe that by using telemedicine, I can easily schedule appointments without having to go to the hospital.” (Participant 1221)*

## Discussion

The study determined the level of acceptance of telemedicine compared to in-person visits, identified the perceived advantages of telemedicine over in-person visits, and explored the reasons why patients choose either of these two types of visits. In the following, the findings of this study are discussed.

### Comparison with previous studies

Most favored in-person visits, while those opting for telemedicine had PhDs, skin/hair/neuropsychiatric conditions, disabilities, or prior telemedicine experience. Telemedicine’s benefits included cost reduction, infection control, distance elimination, and time efficiency. In-person visits were chosen for precise diagnosis, examination, and treatment, while telemedicine was preferred for lower costs, infection prevention, and time savings.

In contrast to our study, where in-person visits were favored, Moulaei et al. [8], preferred to use tele-pharmacy services rather than visiting the pharmacy in person. Similar to our findings, patients chose tele-pharmacy primarily to “reduce costs,” “save time,” and “lower disease transmission risk,” while in-person visits were driven by factors like “direct pharmacist communication,” “limited internet access,” and “addressing patient anxiety and enhancing peace of mind”. Non-acceptance of the use of telemedicine by patients was observed while the use of this technology has expanded significantly in the last decade. Kichloo et al. [35] noted that the scope of telemedicine has rapidly expanded worldwide, connecting global providers and patients to address physician shortages, distribute medical resources more equitably, and enable distinct pathways for specialized care. [35]. Other studies [36–38] highlight that telemedicine offers convenient access to care providers globally, reducing travel expenses and inconvenience, particularly when used as an alternative for costly services or over long distances from medical centers. But it’s important for us to understand that widespread acceptance and high satisfaction among patients and healthcare providers are crucial for successful telemedicine. This necessitates involvement in system design, administrative support, ensuring reliability, user-friendliness, and fair reimbursement [36]. Physician satisfaction drives adoption; education, patient training, and online support improve acceptance [39]. Ultimately, telemedicine must provide value, convenience, and satisfaction akin to in-person visits [36].

In our study, patients also favored telemedicine to overcome distance barriers, decrease extra trips, enhance treatment adherence, and minimize waiting times. Other studies have also shown that telemedicine can reduce the time away from work or school, reduce the waiting time of patients, increase the number of visits made by specialists [40] and easy access of patients living in deprived areas and rural and urban communities to therapists by reducing and remove the geographical distance [41]. A study by Nguyen et al. [36] also indicated that reduced travel time and costs elevate patient willingness to use telemedicine. Consequently, patient and therapist contentment with telemedicine involves diverse aspects beyond health, encompassing home comfort, care continuity, accessibility, cost savings, and convenience. [36]. So, when designing a telemedicine system, not only the dimensions of treatment or health should be considered, but also the other dimensions introduced should be included.

In our study, patients leaned towards telemedicine for treatment to reducing the possibility of contracting infectious diseases, employ effective contact-free evaluations, protect service providers, and prevent disease spread. Alexander et al. [42] asserted that the unexpected outbreak of COVID-19 has reshaped people’s perceptions of telemedicine. In this context, telemedicine can play a vital role in conserving healthcare resources, such as personal protective equipment, while ensuring the delivery of safe and high-quality patient care. Furthermore, it facilitates the maintenance of social distancing measures, thus aiding in the minimization of virus transmission. The findings from the study conducted by Ben-Ar et al. [43] demonstrated a noteworthy surge in the utilization of telemedicine visits for managing upper extremity musculoskeletal complaints within outpatient settings during the COVID-19 pandemic. The study reported a staggering 76-fold increase in telemedicine visits within a specific institution. It is important to emphasize that, prior to the COVID-19 pandemic, the adoption of telemedicine services remained limited across various medical disciplines, despite the documented advantages for patients. These advantages encompassed reduced time spent in the office, alleviated travel burdens and associated costs, heightened patient comfort, and the opportunity for family members to participate in medical consultations[11, 44]. Therefore, it can be said that the significant increase in the use of telemedicine services during the COVID-19 epidemic could be due to the limited access to in-person visits (most physicians’ offices were closed during the epidemic) and the fear of contracting COVID-19 through in-person visits [43]. It is noteworthy to highlight the significance of endorsing telemedicine during a pandemic. This endorsement serves as a mechanism to avert direct exposure of individuals to infectious diseases while surmounting the many barriers that obstruct access to care within clinical settings [45].

Moreover, our study’s findings indicate that in-person visits offer advantages like improved clinical history, direct and comfortable patient-physician communication, focused physician attention, strong patient-physician rapport, and effective treatment guidance. Research by Gordon et al. [46] highlighted issues with clinical video telehealth (CVT) encounters, including provider inattentiveness due to computer monitor and note-related distractions, leading patients to feel overlooked and unheard. Studies emphasize that in-person visits’ success is tied to factors such as friendly interactions, information exchange, body language, and face-to-face engagement, positively impacting patient satisfaction [9, 36]. Non-verbal cues like eye contact are vital in communication, but telemedicine’s camera view limits these cues, creating a challenge [47]. Overcoming this entails training patients to engage actively and teaching providers to eliminate distracting behaviors and use literal cues when addressing the camera [46, 48].

### Study implications

The study’s findings have significant implications for integrating and adopting telemedicine in healthcare. Despite telemedicine’s potential to overcome barriers and provide efficient medical services, patients still prefer in-person consultations. Factors affecting this preference, like education, health conditions, and experience, highlight the need for tailored approaches to promote telemedicine. Addressing these factors could make telemedicine more appealing to a broader patient group. Additionally, the study highlights perceived advantages of telemedicine over in-person visits, such as cost savings, disease prevention, distance elimination, and time-saving. Leveraging these benefits could form the basis for targeted marketing and awareness efforts, emphasizing telemedicine’s tangible benefits. The study’s main conclusion is that despite telemedicine’s advantages, patients still favor in-person visits, underlining the importance of effective change management in healthcare. To encourage wider telemedicine acceptance, healthcare institutions must align with patient preferences and concerns, requiring a strategic approach to address worries and foster an open mindset.

### Study limitations and strengths

The study emphasizes key strengths in its design and findings. Firstly, it centers on patients’ perspectives and preferences concerning Telemedicine vs. in-person visits, augmenting our understanding of patient behavior and decision-making in the evolving healthcare technology landscape. Secondly, the mix of quantitative and qualitative approaches enhances analysis depth, enabling nuanced exploration of patient preferences. The substantial sample size, 1226 participants out of 2059 invited, enhances statistical validity and potential findings’ generalizability. The study also identifies and categorizes influential factors in telemedicine preference, such as education, health status, and prior experience. This showcases its ability to offer actionable insights for healthcare providers to effectively customize services. Additionally, the qualitative analysis of participant responses enriches findings by capturing diverse perspectives and reasons for patient choices.

Lastly, the study’s conclusions and recommendations hold value for the telemedicine field. By recognizing patients’ preference for in-person visits and endorsing strategic change management, the study offers practical guidance to promote broader acceptance and adoption of telemedicine services within the population. Furthermore, in light of the study’s results, healthcare institutions should prioritize continuous patient education and awareness campaigns. These campaigns can help demystify telemedicine, elucidate its benefits, and dispel common misconceptions. By providing accessible and clear information to patients, healthcare providers can empower them to make informed choices regarding telemedicine, ultimately leading to greater acceptance. Additionally, as demonstrated by the findings of this study, fostering strong patient-provider relationships within the telemedicine context is crucial. Healthcare professionals should actively engage with patients, ensuring personalized care experiences. This can be achieved through regular follow-up calls, virtual check-ins, and a commitment to understanding each patient’s unique needs. Building trust and rapport in telemedicine interactions can significantly improve patient satisfaction and acceptance. Finally, technological accessibility remains a barrier for some patients. To address this, healthcare institutions can explore partnerships with local community centers and organizations to provide access to telemedicine technology and resources. By extending telehealth services to underserved populations and offering support for digital literacy, healthcare providers can help bridge the digital divide and broaden telemedicine adoption.

Additionally, this study had limitations. Our survey was exclusively conducted with the participation of patients referred to medical centers and hospitals in four provinces of Iran. Consequently, the outcomes of this study might not be broadly applicable to other countries. Hence, further extensive research is warranted. Furthermore, the study only involved patients, whereas future investigations are advised to encompass both healthy individuals and healthcare service providers. A limitation of our study, attributed to the Covid-19 pandemic, is the predominant utilization of social media for questionnaire distribution, potentially introducing selection bias by excluding those lacking mobile phones or social media engagement. To mitigate this, forthcoming studies could diversify their distribution strategies, employing methods like face-to-face interviews or surveys to encompass individuals with limited online access. Another limitation of our study was that we did not employ established models like UTAUT or TAM. Future research could explore the application of these models and constructs to provide a more comprehensive analysis of the factors influencing telemedicine adoption and acceptance. Other limitation of this study was the potential for selection bias, as participants volunteered to participate. To address this limitation, it is advisable to conduct a follow-up study with a more diverse and randomized sample, which can help mitigate potential selection bias.

## Conclusion

The study compared patient preferences for telemedicine and in-person visits. Despite telemedicine advancements, most still preferred in-person appointments. Common reasons for in-person choices included “More accurate diagnosis of the disease”, “More accurate and better examination of the patient by the physician” and “More accurate and better treatment of the disease”. For telemedicine, top reasons were “Reducing infectious diseases and preventing their further spread”, “Reducing treatment costs” and “Saving time”. Key telemedicine advantages included avoiding infectious diseases, saving cost, and eliminating and overcoming geographical distance barriers.

The reasons and advantages identified in this study can offer opportunities for policymakers, governments, healthcare authorities, and health planners to reshape patients’ negative perceptions and perspectives on telemedicine. The ongoing and growing utilization of telemedicine relies on patients’ and therapists’ acceptance of this healthcare service model. Furthermore, the insights from this study can guide designers and general managers of telemedicine systems in overcoming obstacles and challenges tied to patient non-acceptance. This can lead to more successful design and implementation outcomes.

### Supplementary Information

**Additional file1. **Patients’ perspectives and preferences toward telemedicine versus in-person visits.

## Data Availability

The datasets generated and/or analysed during the current study are not publicly available (due to compromising the privacy of individuals) but are available from the corresponding author on reasonable request.

## Abbreviation

CVT: Clinical video telehealth
