# Supplementary material for: Patients’ perspectives and preferences toward telemedicine versus in-person visits: a mixed-methods study on 1226 patients
Source: BMC Med Inform Decis Mak. 2023 Nov 15;23:261. doi: 10.1186/s12911-023-02348-4 (PMC10647122; doi:10.1186/s12911-023-02348-4)
Supplement: Supplementary file 1 — Additional file1. Patients’ perspectives and preferences toward telemedicine versus in-person visits. [file 12911_2023_2348_MOESM1_ESM.docx]

**Additional file 1**

**Patients' perspectives and preferences toward telemedicine versus in-person visits**

Dear participant, this questionnaire has been developed to determine the patients' perspectives and preferences toward telemedicine versus in-person visits. Please answer each question after reading it.

***Section A: Demographic and clinical characteristics***

1. **What is your age in years? .........**
2. **What is your gender?**

- Female
- Male
- Trans-Female
- Trans-Male

1. **What is your education level?**

- Diploma
- Associate
- Bachelor
- Master
- PhD and higher

1. **What type of your residence?**

- Metropolitan areas
- Remote and rural areas

***Section B: Medical Conditions***

1. **What type of disease do you have?**

- Oral and dental diseases
- Eye diseases
- Skin and hair diseases
- Digestive diseases
- Otorhinolaryngology diseases
- Gynecological diseases
- Endocrine diseases
- Psychological disorders
- Musculoskeletal diseases
- Cardiovascular diseases
- Respiratory diseases
- Cancers
- Rheumatology diseases
- Kidneys and urinary diseases
- Genetic disorders
- Infectious diseases
- Birth Defects and disabilities
- Rare diseases
- Chemotherapy

1. **How long have you had the disease (year)?**

- 1-10
- 11-20
- >=21

1. **Do you have experience of using telemedicine for treatment?**

- Yes
- No

***Section C: Advantages of using telemedicine versus in-person visit***

| **Row** | **Advantages of using telemedicine versus in-person visit** | **Strongly Disagree** | **Disagree** | **Undecided** | **Agree** | **Strongly Agree** |
| --- | --- | --- | --- | --- | --- | --- |
|  | Better patient-therapist interaction |  |  |  |  |  |
|  | Easy exchange of information between the patient and the therapist |  |  |  |  |  |
|  | More patient peace of mind for treatment |  |  |  |  |  |
|  | Saving costs |  |  |  |  |  |
|  | Schedule the next visit |  |  |  |  |  |
|  | Easy electronic collection and storage of medical data and information |  |  |  |  |  |
|  | Getting better treatment advice |  |  |  |  |  |
|  | Quick diagnosis of the disease |  |  |  |  |  |
|  | Less embarrassment and shame of the patient in providing information to the therapist |  |  |  |  |  |
|  | Reduction of medical errors |  |  |  |  |  |
|  | Avoiding infectious diseases |  |  |  |  |  |
|  | Improving lifestyle |  |  |  |  |  |
|  | Increasing the patient's self-confidence in performing treatment processes |  |  |  |  |  |
|  | Convenient sharing of knowledge and information between therapists when faced with a rare disease |  |  |  |  |  |
|  | Eliminate and overcome geographical distance barriers |  |  |  |  |  |
|  | Easy reporting of drug and treatment side effects to the physician |  |  |  |  |  |
|  | Easy presentation of the patient's treatment history to the therapist |  |  |  |  |  |
|  | Better presentation of the patient's current history to the therapist |  |  |  |  |  |
|  | Better and more accurate medicine prescription |  |  |  |  |  |
|  | Better and easier follow-up of treatment instructions |  |  |  |  |  |
|  | Better and easier use of health insurance |  |  |  |  |  |
|  | Greater honesty of the patient in providing information to the therapist |  |  |  |  |  |
|  | Professional commitment of the therapist to treat the patient |  |  |  |  |  |
|  | Maintaining confidentiality and privacy of information |  |  |  |  |  |
|  | Easy follow-up of medical malpractices |  |  |  |  |  |
|  | Giving more time to the therapist to treat the patient |  |  |  |  |  |
|  | Faster recovery of the patient |  |  |  |  |  |
|  | Easily perform self-care and self-management processes |  |  |  |  |  |
|  | Making better and more accurate treatment decisions by the therapist |  |  |  |  |  |
|  | Receive quality medical services |  |  |  |  |  |
|  | Greater medication adherence |  |  |  |  |  |
|  | Spending less time to receive medical services |  |  |  |  |  |
|  | Better interpretation of laboratory tests |  |  |  |  |  |
|  | Easy to pay medical bills |  |  |  |  |  |
|  | Better diagnosis of the disease |  |  |  |  |  |
|  | Reducing patient anxiety and stress |  |  |  |  |  |

***Section D: Your preference for treatment procedures***

1. **What is your preference?**

- Telemedicine
- In-person visits

1. **Explain the reasons for choosing telemedicine or in-person visits?**

**……………………………………………………………………………………………………………………………………………………………………………………………………………………………………………………………………………………………………………………………………………………………………………………………………………………………………………………………………………………………………………………………………………………………………………………………………………………………………………………………….**
